# Exploring hypermasculinity as a moderator between sexual violence victimization and adverse mental health effects among sentenced incarcerated men

**DOI:** 10.22514/jomh.2024.169

**Published:** 2024-10-30

**Authors:** Sebenzile Nkosi, Kopano Ratele, Yandisa Sikweyiya, Neo Morojele

**Affiliations:** 1 Mental Health, Alcohol, Substance Use and Tobacco Research Unit, South African Medical Research Council, 0084 Pretoria, South Africa; 2 Department of Psychology, Rhodes University, 6140 Makhanda, South Africa; 3 Department of Psychology, University of Johannesburg, 2092 Johannesburg, South Africa; 4 Department of Psychology, Stellenbosch University, 7600 Cape Town, South Africa; 5 Gender and Health Research Unit, South African Medical Research Council, 0084 Pretoria, South Africa; 6 School of Public Health, Faculty of Health Sciences, University of the Witwatersrand, 2017 Johannesburg, South Africa; 7 School of Family Medicine and Public Health, Faculty of Health Sciences, University of Cape Town, 7700 Rondebosch, South Africa

**Keywords:** Incarceration, Hypermasculinity, Sexual violence, Prototypic depression, Masculine depression, Alcohol and other drug use

## Abstract

Sexual assault is a common occurrence among incarcerated men in correctional facilities, and severely impacts the psychological well-being of the victim. We explored hypermasculinity as a moderator between sexual violence (SV) victimization and consequent mental health outcomes (prototypic and masculine depression, alcohol use and drug use) among incarcerated men in Tshwane, South Africa. A convenience sample of 160 incarcerated men self-completed two questionnaires: Questionnaire 1, completed during their incarceration, included themes such as demographics, incarceration-related characteristics, and involvement in physical fighting, and Questionnaire 2, completed at least two months post their release from incarceration, included themes such as demographics, gang affiliation, hypermasculinity, SV victimization, misuse of substances and/or alcohol, prototypic depression and masculine depression. Descriptive analyses were conducted to examine sample characteristics. Associations between variables were examined with Pearson’s correlational analysis while the moderation effect was tested with the SPSS PROCESS macro software. Hypermasculinity had a significant moderation effect on SV victimization and prototypic depression. *Post hoc* probing analyses showed a significant negative relationship between SV victimization and prototypic depression among men who highly endorsed hypermasculinity, but no significant relationship was observed among those who endorsed hypermasculinity at low or average levels. The moderation effect of hypermasculinity was not statistically significant for SV victimization and masculine depression, alcohol use and drug use. Men who were high in hypermasculinity had a decreased likelihood of prototypic depression despite having been sexually victimized. Interventions that address SV-related mental health outcomes should take into account the role of masculine ideals in SV-related psychological reactions among incarcerated men or men with a history of incarceration.

## 1. Introduction

Sexual violence (SV) victimization has deleterious consequences on the psychological health of survivors, including their levels of depression and substance misuse [1–3]. Incarcerated men have a heightened risk of exposure to SV victimization within correctional facility settings, with studies reporting prevalence rates of SV victimization as high as 26% [4–6], and potentially even higher rates in South Africa [7, 8]. Masculinity norms play a significant role in how men make meaning of their SV victimization experiences [9–12]. Perceptions of having failed as a man and feelings of self-blame and emasculation are common among male SV survivors [10, 11, 13, 14]. These feelings and perceptions are potentially worse for men who experience SV in correctional facility settings given the overt use of sexual dominance to establish and maintain masculinity hierarchies in these settings [9, 15].

### 1.1 Sexual violence among incarcerated men in South Africa

Few studies have examined SV among men in correctional centers in South Africa [8, 9], as is the case globally [16]. From their exploration and description of SV, the few studies that do exist have highlighted gendered power struggles among men as a significant contributor to the assertion of dominance of the perpetrator over victims in correctional centers [9]. Young, first-time incarcerated men are typically targeted and tricked into exchanging items such as cigarettes, drugs and food for sex and/or a sexual relationship. Providers of these items often demand sex from the recipient. A common outcome for recipients includes rape and/or being forced into a sexual pairing [9].

Relatedly, prison gangs also contribute significantly to violence in South African correctional centers. In particular, the 28 Numbers Gang specifically encourages sexual relationships (often coerced or forced) among its members in correctional centres and has, within its structure, what it refers to as a “gold line” or soldiers (men who retain a masculine status) and a “silver line” (men who are feminized). Often, “silver line” members are tricked and/or forced into sexual relationships [17]. Notably, however, despite the endorsement of sexual violence by the 28s gang, a prevalence study on sexual violence in a juvenile correctional center suggests that a notable proportion of violence occurs outside of gangs [18]. Thus, research on violence in correctional centers requires a focus that is inclusive of the wider population of incarcerated men. Moreover, previous descriptive, exploratory work provides a foundation from which to quantitatively test the role of masculinity in men’s experiences of SV.

### 1.2 Men and depression

The prevalence of depression among men is half that of women, globally [19]. This gap appears even larger among incarcerated people in South Africa as demonstrated in a study with 96% male participants which found the lifetime prevalence of depression to be 24.5% [20], while a study with incarcerated women reported a 70% prevalence of depression [21]. However, the prevalence gap between incarcerated men (9.5%) and incarcerated women (15.4%) for current depression in these studies was smaller than compared to the general population [20, 21]. Men’s lower propensity towards depression is more a matter of societal expectations towards men than it is a matter of vulnerability [22]. The higher rate of suicide among men compared to women globally is often used to support this view [19, 23]. In South Africa, studies show that suicide prevalence among men are 3–4 times the rate observed among women [19, 24, 25].

Gender norms, or society’s expectations of how men and women should behave, contribute to the differential prevalence of depression between men and women [26]. Prototypic depression, the variant of depression captured in diagnostic tools such as the Diagnostic and Statistical Manual (DSM), with symptoms that include low mood, sadness, hopelessness and social withdrawal, is arguably misaligned with society’s expectations of men or masculinity norms [27]. Rather, men are generally expected to demonstrate toughness, self-control, emotional restriction and risk-taking [26–29] which, in situations of psychological distress, tend to manifest as avoidance coping, risk-taking behavior, substance use, aggression, self-harm and suicide [25, 29].

“Masked depression” and “masculine depression” are concepts used to explain the phenomenon of depression commonly observed among men [27]. The concept of masked depression suggests that men respond to distress in ways that are aligned with masculinity norms and therefore conceal their experience of distress by suppressing their emotional distress and/or expressing it in externalizing ways such as aggression [28]. The concept of masculine depression suggests that men’s internalization of masculinity norms renders their experience of distress different to the way in which women experience it, resulting in a different variant of depression altogether [22, 28].

### 1.3 Men and substance use disorders

With the exception of prescription or over-the-counter medication, men are disproportionately more likely to (mis)use alcohol and other substances and develop dependence, as compared to women [30, 31]. In a recent South African national prevalence study, men were almost four times (16.5%) as likely to consume alcohol at hazardous, harmful, and dependent levels than women (4.6%) [32]. The highly prevalent, problematic use of alcohol is reflected in its being the most common substance for which people seek treatment in South Africa [33]. Similarly, other drug use is more prevalent among men than women, and its use has increased substantially over time in South Africa [33, 34]. Moreover, despite being prohibited, alcohol and other drug use is prevalent in correctional settings. For example, substance use disorder was the most prevalent mental disorder (42%) among incarcerated people in a point prevalence study of mental disorders in KwaZulu Natal, conducted in 2009 [20].

Substance use is associated with avoidance coping against unpleasant emotions and adversities [32]. Avoidance coping strategies are more prevalent among men than women, because they enable numbing and/or restriction of displays of vulnerability, such as grief, sadness and fear that could compromise men’s sense of self, and displays of being stoic and in control [22]. In situations of adversity, behaviors that are deemed more masculine, such as substance use, may find expression among men [26].

### 1.4 Theory of masculinity

Literature on men’s gendered response to psychological distress is limited by its presentation of men as a unified group, with comparisons generally made between men and women and rarely within the category of men [35]. Connell’s [36] pioneering concept of hegemonic masculinity highlights the importance of examining differences as a function of masculinity relations among men. Connell [36] theorized that masculinities, or notions of what it means to be a man, are multiple. She identified four hierarchical masculinity positions that are generally in operation in a given context. Hegemonic masculinity, the culturally endorsed form of masculinity within a social context, sits at the very top of any masculinity hierarchy and enjoys, as well as legitimizes, power and authority of men over women [36]. Access to power and privileges incentivizes most men to aspire to hegemonic masculinity, although most men who aspire to it never achieve it and some men actively resist it [36]. The majority of men tend to be situated within complicit, marginalized, or subordinated masculinity positions [36]. Complicit masculinities, are occupied by men who derive benefits from hegemonic masculinity and, although they may not explicitly enact its practices, they are invested in it, do not challenge it, and may defend it [36]. Marginalized masculinities are occupied by individuals who “lack” the attributes that would make hegemonic ideals attainable. Poor, black men often occupy these positions as hegemonic masculinities often equate to privileged attributes, which generally include being white and having access to wealth. Subordinate masculinities are occupied by individuals who exhibit oppositional qualities that challenge hegemonic ideals, for example, men who have sex with men [36].

When hegemonic masculinity ideals are unattainable, *e.g*., for poor black men in contexts where hegemonic masculinity ideals equate to whiteness and wealth, some men can appropriate hypermasculinity as a form of resistance against the exalted form of masculinity [37]. Hypermasculinity is a maladaptive form of masculinity in which attributes such as displays of invulnerability, hardness and bravado are exaggerated [37]. This form of masculinity has been found to be dominant in correctional facilities, owing to the importation of deviant practices by the incarcerated population as well as exaggerated competition among men due to deprivations of the environment such as scarcity of material resources [38]. In correctional centers, hypermasculinity is typically performed through displays of toughness, aggression, and violence against other men who are perceived as weak and vulnerable [38, 39].

In this study we sought to explore whether and how hypermasculinity moderates the relationship between experiencing SV and the adverse mental health effects amongst sentenced incarcerated men. This study will contribute to the limited body of research on men’s psychological responses to SV victimization [3], and particularly, the few quantitative studies among incarcerated men in South Africa. We expected elevated levels of internalizing mental health outcomes (*i.e*., prototypic depression) among men reporting low hypermasculinity and elevated levels of externalizing mental health outcomes (*i.e*., masculine depression, alcohol use and drug use) among those reporting high hypermasculinity. Specifically, we explored the following hypotheses:

Hypothesis 1a: There will be a positive relationship between SV victimization and prototypic depression in men who are low in hypermasculinity and a negative relationship in men who are high in hypermasculinity.

Hypothesis 1b: There will be a negative relationship between SV victimization and masculine depression in men who are low in hypermasculinity and a positive relationship in men who are high in hypermasculinity.

Hypothesis 2: There will be a negative relationship between SV victimization and alcohol use and drug use in men who are low in hypermasculinity and a positive relationship in men who are high in hypermasculinity.

## 2. Materials and methods

### 2.1 Research setting

The research was carried out in the City of Tshwane in Gauteng, South Africa. According to current publicly available official statistics, Gauteng has approximately 25,569 detained people, of whom 24,694 are men [40]. Tshwane region correctional facilities house about 31% of Gauteng’s sentenced incarcerated population [41]. Correctional facilities across the country have a chronic overcrowding issue, currently shown at about 129% capacity at centers that detain adult men [42]. Overcrowding carries adverse security implications as it translates to a compromised ratio of correctional officers to the incarcerated population. In South Africa, the ratio of correctional officers to incarcerated people is 1:9 instead of the recommended 1:5 [40, 42].

### 2.2 Research design

This paper presents cross-sectional quantitative data from a larger mixed methods investigation. The original study employed a parallel explanatory design with a dominant quantitative component to explore the role of hypermasculinity as a predictor of SV perpetration and victimization and a moderator of the relationship between perpetrating or experiencing SV and adverse mental health effects. The quantitative component entailed a two-part survey that was administered during and post the incarceration of the participant. The survey was administered in two parts to enable assessment of change in hypermasculinity during incarceration compared to post incarceration (a research question not covered in this paper). However, most of the measures were not repeated in order to reduce the burden of questionnaire completion for participants (*i.e*., to keep interviews to a maximum of 60 minutes).

### 2.3 Participant recruitment and data collection

Participants were recruited from detention correctional facilities during their period of incarceration through convenience sampling from August 2016 to October 2017. Researchers (first author and trained research assistants) visited six detention correctional facilities to recruit participants. Correctional officers announced the researchers’ visits to the men detained at the facilities and escorted those who were interested in the study to the researchers who were situated in rooms designated for the research activities within the facility. Following further briefing by the researchers, and assessment of eligibility—based on the following: age 18 years and above, ≥grade 8 education, fluency in English or Setswana, parole date (actual or estimated) was within the next six months, and parole supervision would be at a community correctional facility within Tshwane—interested and eligible prospective participants completed informed consent forms. In addition, they provided contact information of two to three family members and/or friends with whom they planned to stay in regular contact after their release from incarceration. Participants then completed a self-administered questionnaire (*i.e*., Questionnaire 1).

Participants were contacted telephonically to participate in a second survey at least two months post their release. The follow-up study procedures were carried out on a day that coincided with a compulsory monthly visit at the community correctional facility where the participant was undergoing correctional supervision. Participants were asked to complete a second informed consent form and another self-administered questionnaire (*i.e*., Questionnaire 2).

### 2.4 Instruments

Participants had the option of completing the questionnaire in English or Setswana. Translation of the study’s questionnaires from English to Setswana followed a process similar to the one described by Sousa and Rojjanasrirat [43]. Setswana language speakers who were fluent in English, lived in the Tshwane region, and were familiar with the cultural nuances of the Setswana language of the region translated the materials. The translators worked in sequence and independently of each other. One of the translators translated the questionnaire from English to Setswana and the other translator translated the Setswana questionnaire to English. The first author reviewed the back-translated (English) version against the original (English) questionnaire using the compare function in Microsoft Word and noted any discrepancies in meaning. The discrepancies were discussed and resolved via consensus with both translators, where possible. Where disagreements persisted between the translators, a third translator, who was a native speaker of Setswana, and an experienced public health researcher and clinical psychologist, was consulted to review the parts on which there was disagreement. The third reviewer’s comments and suggestions were discussed with the translators until consensus was reached.

We conducted a pilot study to test our measures prior to conducting our main study. The during-incarceration measures were tested among sentenced incarcerated men and the post-incarceration measures were tested among men who were undergoing parole supervision. All multi-item measures reached acceptable internal consistency reliability, *i.e*., ≥0.70. Reliability analyses were also conducted with the main study’s data and are reported here with the description of the present study’s measures, where applicable.

#### 2.4.1 Questionnaire 1

This questionnaire was administered via paper and pen.

Demographic characteristics and information relating to incarceration: the questionnaire solicited information about the participants’ education, relationship status, number of years they had been incarcerated, the crime for which they were convicted, previous convictions and their involvement in physical fights.

#### 2.4.2 Questionnaire 2

This questionnaire was administered via electronic handheld devices.

Demographic characteristics and information relating to incarceration: the questionnaire solicited information about the participants’ age and whether they had been part of a prison gang during their latest period of incarceration.

Hypermasculinity was assessed with the Auburn Differential Masculinity Inventory (ADMI), which measures a respondent’s endorsement of hypermasculinity. The ADMI is a Likert scale comprising 60 items. Its response options range from 1 (very much like me) to 5 (not at all like me). A sample item extracted from this measure is “I think men who show they are afraid are weak”. The total score is the sum of the individual item scores, which ranges from 60 to 300. Lower scores on this measure denote a high endorsement of hypermasculinity [44]. The internal consistency reliability for the measure was 0.91 in this study.

The tool used to assess sexual violence victimization was created using an adapted version of the sexual victimization measure by Wolff *et al*. [45]. Five of the items of Wolff *et al*.’s [45] measure were adapted from the United States’ National Violence Against Women (NVAW) survey; these ask about completed, threatened, and attempted sexual acts by a fellow inmate and/or staff member. An additional five items elicit information about abusive sexual acts (three items, *e.g*., has another inmate/staff member touched you, felt you or grabbed you in a way that you felt was sexually threatening?) and coercion (two items, *e.g*., has another inmate/staff member required you to perform acts of a sexual nature in exchange for protection from future harm?). In developing their measure, Wolff *et al*. [46] sought to create a comprehensive SV measure that would align with a SV definition that encompasses a range of non-consensual sexual acts, including forced or threatened sexual acts (vaginal, oral and anal sex), and abusive sexual contacts such as touching of specific areas of the body. The following adaptions were made to Wolff *et al*.’s [45] measure for this study: (i) we deleted a question on “made to have sex” which, in light of types of sexual acts being generally specified in the questions (oral and anal), we interpreted unspecified sex to mean vaginal sex thus not applicable for our sample, (ii) we added a question about “made to have thigh sex” as qualitative literature has shown that this sexual act is prevalent in male South African correctional facilities [13], (iii) we used a reflection period of 12 months (during incarceration; similar to NVAW) rather than 6 months as per Wolff *et al*. [45], (iv) we changed response options from a binary “yes” or “no” response to a 4-point Likert scale format of 0 (never), 1 (only once), 2 (more than once by 1 person) to 3 (more than once by 2 or more people), and (v) we only asked about inmate violence. The measure’s internal consistency was 0.71.

Alcohol consumption was assessed with the Alcohol Use Disorders Identification Test (AUDIT) scale. The AUDIT is a 10-item measure that elicits information on an individual’s frequency and quantity of alcohol consumption and experiences of alcohol-related problems. Item scores are summed to get a total score that can range between 0 and 40. A score of 8 or higher indicates at-risk alcohol consumption. The AUDIT has been employed in studies across various regions of the world, including South Africa [47, 48]. Its internal consistency reliability was 0.85.

Drug use was assessed with the Drug Use Disorders Identification Test (DUDIT). The DUDIT is an 11-item measure that elicits information on an individual’s frequency and pattern of drug-use. Item scores are summed to get a total score of between 0 and 44. A total score of 6 or higher denotes problem drug use [49]. This measure has been used among various populations and settings, including incarcerated people and South Africa, respectively [50, 51]. Its internal consistency reliability was 0.80.

Prototypic and masculine depression were assessed with the Masculine Depression Scale (MDS). The MDS tool consists of Likert scale items that make up two subscales: internalizing symptoms, which characterize prototypical symptoms, and externalizing symptoms, which characterize “acting out” behaviors linked to normative masculinities [52]. Sample items for the internalizing and externalizing subscales, respectively, are “I have yelled at people or things” and “I don’t feel as powerful”. There were five possible responses, which ranged from 1 for none or only occasionally, to 5 for always. Internal consistency reliability was 0.95 for the entire measure, 0.95 for the internalizing subscale and 0.75 for the externalizing subscale.

### 2.5 Sample size

A sample size calculation was conducted on nQuery (version 7, Statsols, Cork, Ireland). The sample size is given by

$$n=\frac{{\left[z_{1-\alpha /2}\sqrt{\pi_{0}\left(1-\pi_{0}\right)}+z_{1-\beta }\sqrt{\pi_{1}\left(1-\pi_{1}\right)}\right]}^{2}}{{\left(\pi_{0}-\pi_{1}\right)}^{2}}$$

Based on a prevalence of sexual violence of 10–20% [6], and a desired statistical power of at least 80%. A one-group *χ*^2^ test with a 0.050 two-sided significance level would have 80% power to detect the difference between the Null hypothesis proportion, *π*_0_, of 0.100, and the Alternative proportion, *π*_1_, of 0.200 when the sample size is 86.

### 2.6 Analysis

Data were analyzed using SPSS (version 26, IBM SPSS Inc., Chicago, IL, USA). The demographic and incarceration-related characteristics of the participants were analyzed using descriptive statistics. Bivariate associations between the predictor (SV victimization), the moderator (hypermasculinity), and the dependent variables (prototypic depression, masculine depression, alcohol use and drug use) were examined using Pearson’s correlation. The moderating effect of hypermasculinity on the relationship between SV victimization and adverse mental health effects was examined with PROCESS macro, a programme designed for carrying out moderation and mediation analyses in SPSS [53]. The variables in the moderation model were mean centered to minimize multi-collinearity. In line with Hayes and Cai’s [54] recommendation, a heteroskedasticity-consistent standard error estimator (HC2) was used to reduce bias that may be introduced by heteroskedastic errors in the data. Bootstrapping was used with the moderation analyses, with 1000 resampling [53]. Bootstrapping was considered more appropriate than a non-bootstrapping method for this study because it yields higher power and better Type I error control and provides a more reliable estimate of indirect effects, all of which are particularly useful for small sample sizes.

For the Pearson’s correlation we report *r* and *p*-values. For the moderation results we first show the *R*-square and *F* change for each model, followed by the coefficient and *p*-value for the interaction between SV victimization and hypermasculinity on each of the outcome variables. We then report the conditional effects of hypermasculinity on the association between SV victimization and the mental health outcomes. We used the Pick-a-Point convention to operationalize low, moderate, and high values as the 16th, 50th and 84th percentile of the hypermasculinity distribution (Hayes, 2018). Values from the 16th percentile and below are considered low, those between the 16th and 84th percentile are considered moderate, and those from the 84th percentile and above are considered high. Interaction plots are shown to display the slopes of conditional effects of hypermasculinity on the association between SV victimization and each of the mental health outcomes for those with low, moderate and high scores on hypermasculinity. Finally, we present the effect, *t* and *p*-values of the conditional effects of SV victimization on the dependent variables at the value of hypermasculinity that is at a significant point of transition [53].

## 3. Results

From 1102 completed screening questionnaires, 193 men met the eligibility criteria for inclusion in the study. Of these, 33 chose not to participate in the study, resulting in 160 men completing Questionnaire 1. At post-incarceration, 67 (42%) men returned to complete Questionnaire 2. Participants did not return for completion of the second questionnaire for numerous reasons: 19% were unreachable via the contact information they provided, 18% no longer wanted to take part, 8% were arrested for a different offense or parole infraction, 7% were still incarcerated at the time of the follow-up, 4% did not yet meet the minimum release-to-participation period and 2% were released to a setting that was located outside the designated research area.

### 3.1 Demographic and incarceration-related characteristics of participants

Table 1 displays the demographic characteristics and incarceration-related factors of the sample. The average age of the sample was 31.7 years (standard deviation (SD) = 8.80), with a range of 18 to 62 years. Over a third (40.3%) had obtained grade 12 and higher in education. Almost two-thirds (64.2%) were single. On average, the participants had served 3.8 years (SD = 4.00) in detention for their sentences.

Over half of the participants (53.7%) were convicted for property-related offenses (including, but not limited to, robbery, theft, and burglary) as well as other offenses (such as fraud, drug possession, contempt of court, and obstructing the course of justice). Over a third (40.3%) had been convicted for other offense(s) prior to the offense(s) for which they were incarcerated. In the previous year, nearly half (44.8%) had been involved in a physical altercation. Just under a third (28.4%) had been part of a gang during their incarceration. About a fifth (20.9%) had experienced SV victimization at least once.

### 3.2 Bivariate associations

Table 2 shows the bivariate relationships between SV victimization, hypermasculinity, and the dependent variables. Prototypic depression (*r* = 0.387; *p* = 0.002) and masculine depression (*r* = 0.286; *p* = 0.023) had a significant relationship with hypermasculinity, but not with SV victimization. None of the other dependent variables had a significant relationship with hypermasculinity nor with SV victimization.

### 3.3 Moderation effects of hypermasculinity on the relationship between SV victimization and adverse mental health

Table 3 displays the regression analysis results of the moderation effect of hypermasculinity on the relationship between SV victimization and mental health outcomes. For hypothesis 1a, the interaction term between hypermasculinity and SV victimization accounted for a significant proportion of the variance in prototypic depression (Δ*R*^2^ = 0.04, Δ*F*(1, 62) = 4.486, *b* = 0.34, *t*(62) = 2.12, *p* = 0.038).

**FIGURE 1.**
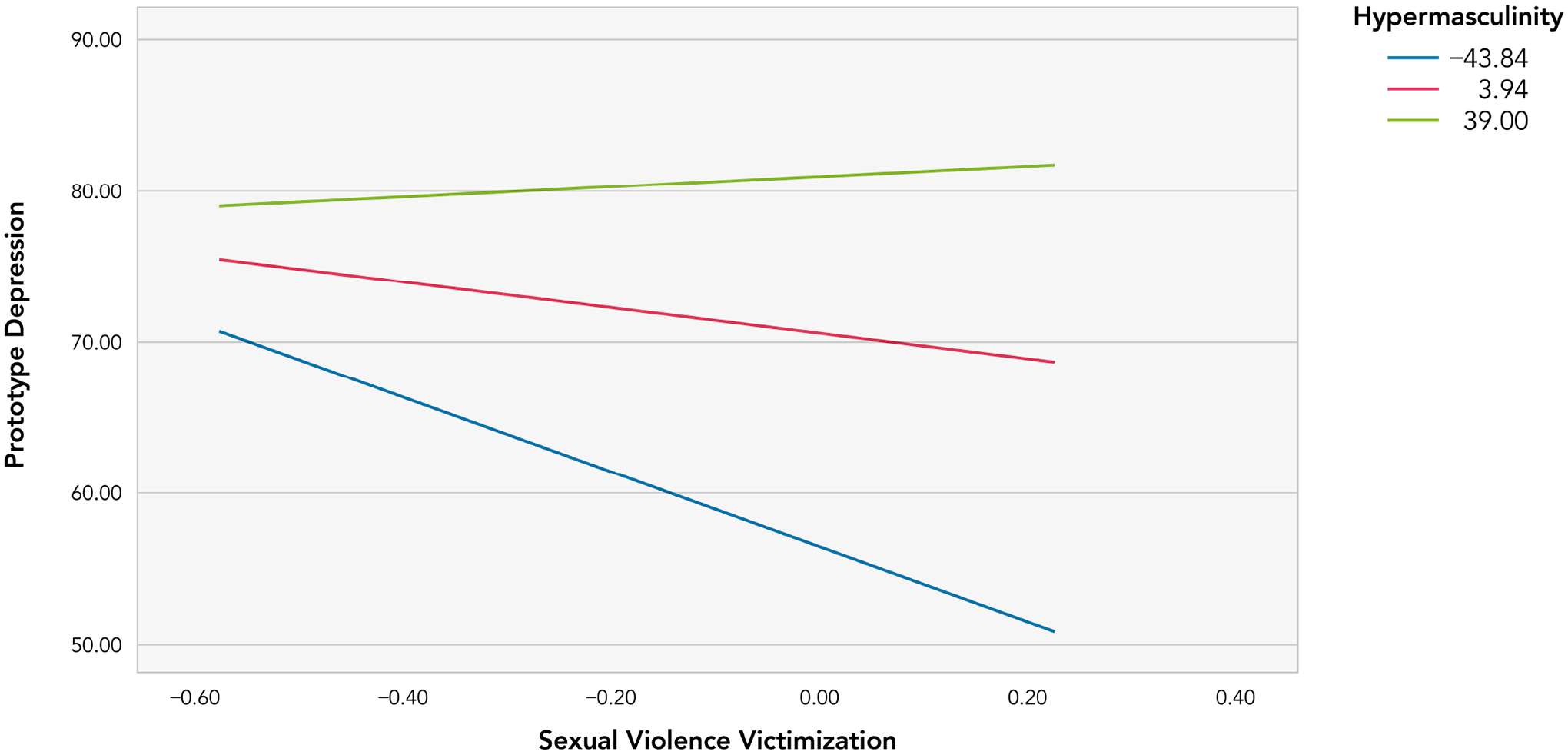
Conditional effects of SV victimization on prototypic depression at the 16th, 50th and 84th percentile of hypermasculinity.

The interaction plot (Fig. 1) shows an effect that increases when hypermasculinity scores decrease. Specifically, at low levels of hypermasculinity, SV victimization had a significantly negative relationship with prototypic depression (Effect = −15.61, *t* = −2.29, *p* = 0.026, 95% Confidence interval (CI): −28.51–−1.92). Prototypic depression and SV victimization had no significant relationship at low or average values of hypermasculinity.

For Hypothesis 1b, the interaction term between hypermasculinity and SV victimization accounted for a significant proportion of the variance in masculine depression (Δ*R*^2^ = 0.05, Δ*F*(1, 61) = 5.96, *b* = 0.11, *t*(61) = 2.44, *p* = 0.019). However, the overall model was not statistically significant (*R*^2^ = 0.14, *p* = 0.053).

**FIGURE 2.**
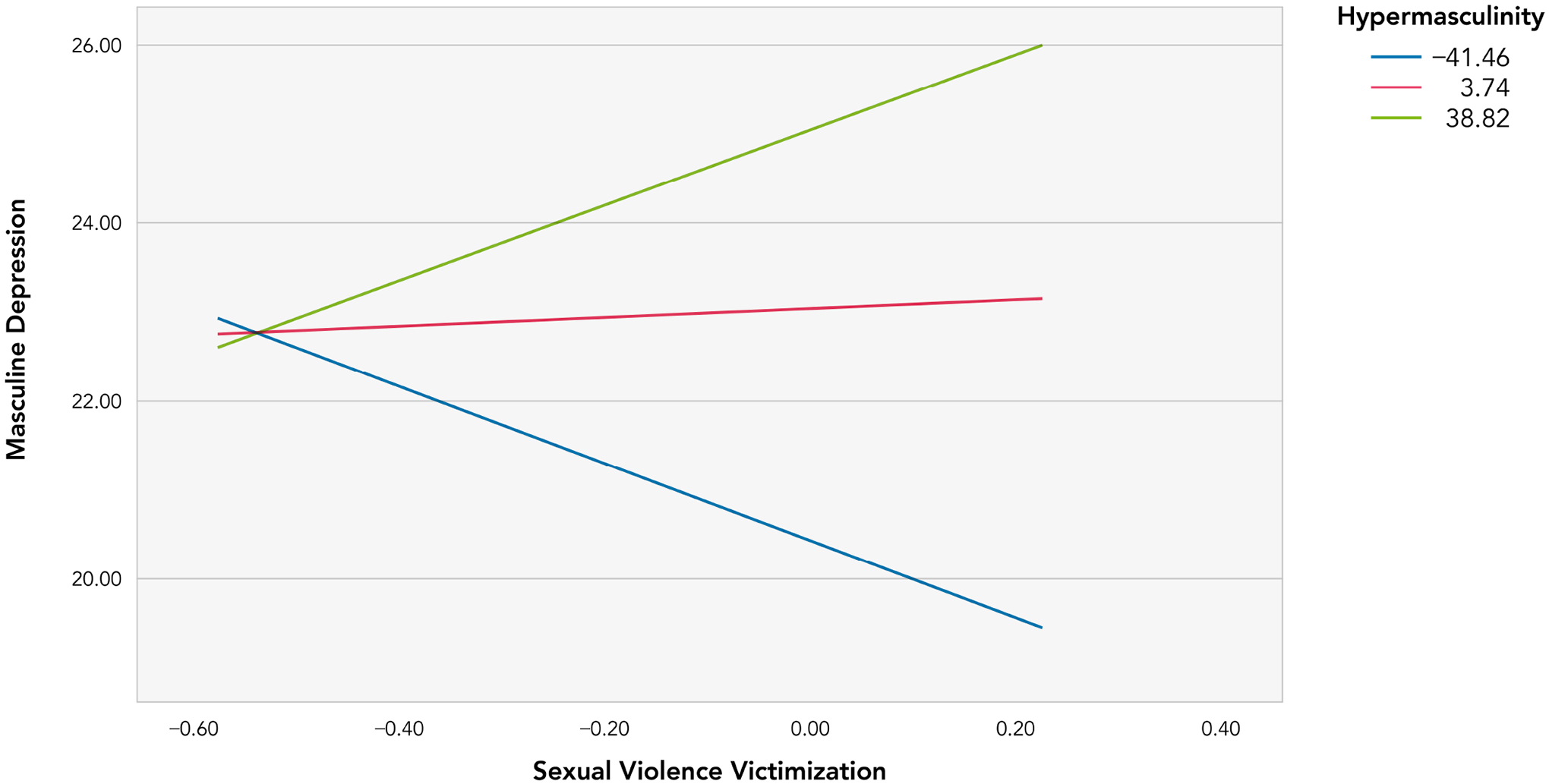
Conditional effects of SV victimization on masculine depression at 16th, 50th and 84th percentile of hypermasculinity.

The interaction plot (Fig. 2) showed an effect that decreases when hypermasculinity scores decrease and an effect that increases when hypermasculinity scores increase. Specifically, at high values of hypermasculinity, SV victimization had a significantly positive relationship with masculine depression (Effect = 3.83, SE = 1.67, *t* = 2.30, *p* = 0.025, 95% CI: 0.49–7.17). Masculine depression and SV victimization did not have a significant relationship at low and average values of hypermasculinity.

With reference to Hypothesis 2, the interaction terms between hypermasculinity and SV victimization were not significant for the AUDIT (*b* = 0.003, *p* = 0.565), and the DUDIT (*b* = −0.004, *p* = 0.720).

## 4. Discussion

The prevalence of SV victimization in the past 12 months among our sample of incarcerated men was about 21%. This is similar to rates reported in some studies [4–6], yet also higher than those reported in other studies of incarcerated populations [46, 55]. Papadakaki *et al*. [5] argue that higher rates of SV victimization often occur in studies with small sample sizes and where a broad definition of SV is used. While the sample size was small, the definition of SV in this study, which included threatening sexual touching and forced penetrative sexual contact, was very similar to definitions used in other studies on SV among incarcerated men [6, 46]. Notwithstanding the high detection benefits of using a broader definition of SV [45], the higher rates of victimization in our study may also be a reflection of the high rates of SV in the larger context of South Africa and the common use of sexual power by some men to attain and maintain dominance over others; a phenomenon that has been particularly observed in relation to male-on-female SV in this setting [56]. Below we discuss the findings for each of our hypotheses regarding the moderation effect of hypermasculinity on the relationship between SV victimization and adverse mental health outcomes (prototypic depression, masculine depression, alcohol use and drug use).

### 4.1 Hypermasculinity, SV victimization and depression

Consistent with our hypothesis, the findings showed that, among men who were high in hypermasculinity, a higher degree of victimization results in a lower likelihood of prototypic depression. This finding is consistent with literature that has uncovered that this form of depression is less likely to be exhibited by men, and in this study, which had an exclusively male sample, it follows that its occurrence would be less likely among those men who are high in hypermasculinity. In line with the theory of masculinity, and that men tend to idealize strength and stoicism, it is plausible that men who are hypermasculine are less inclined to exhibit a depression that is associated with vulnerability [27]. Possibly, the manner in which these men experience and express SV-related psychological distress is via other ways that are different to prototypic depression, such as other mood disorders (*e.g*., mania), anxiety disorders (*e.g*., post-traumatic stress disorder) and/or somatization. These findings may also suggest that these men have heightened resilience towards depression owing to internalization of invulnerability in the face of adversity. Indeed, there is an emphasized research focus on negative aspects of hegemonic masculinities, or their exaggerated variants, which can detract from some of the potential benefits of masculinity norms such as enabling problem-solving focused coping strategies, as highlighted by some scholars [22, 57]. Qualitative research that examines coping with experiences of SV victimization among men who endorse hypermasculinity can help contextualize these findings. Specifically, it would be important to consider whether these findings indicate true resilience or avoidance. Considering that men who endorse hypermasculinity tend to compensate for masculinity ideals that are often unattainable [37], and that sexual violence victimization exacerbates men’s feelings of emasculation [58], our speculation is that they are actively or unconsciously suppressing their distress, and engaging in an avoidance-type coping strategy which can lead to emotional outbursts and violence [59].

Findings for our hypothesis that hypermasculinity has a moderating effect on the relationship between SV victimization and masculine depression were inconclusive. While the moderation effect was significant, the overall model was not significant, possibly due to our small sample size. The conditional effects revealed a significantly negative relationship between SV victimization and masculine depression among men who strongly endorsed hypermasculinity, and a non-significant relationship between SV victimization and masculine depression among men who less strongly endorsed hypermasculinity. These findings are inconsistent with the study’s hypothesis and literature that has theorized and/or found that men who ascribe to normative masculine ideals typically engage in aggressive behavior, use of alcohol, and/or use of other drugs, as these behaviors tend to be within the acceptable repertoire of masculinity [25, 29]. These trends underscore a need for further studies with larger sample sizes.

### 4.2 Hypermasculinity, SV victimization, and alcohol and other drug use

The findings of our study did not support the hypothesis that hypermasculinity moderates the relationship between SV victimization and alcohol and other drug use. The basic associations between SV victimization and alcohol and other drug use were also not significant. These findings are inconsistent with earlier studies that have demonstrated that survivors of SV frequently use alcohol and other drugs as a coping mechanism [2]. In addition, the findings are not in line with literature showing that expectations of men to demonstrate that they are not emotionally vulnerable often result in men’s reliance on emotional-avoidance and numbing strategies, including the use of substances to cope with psychological distress [22]. These negative findings may be explained, in part, by the prohibition of substance use for people who are on parole. According to the Department of Correctional Services guidelines on community correction supervision, people undergoing parole supervision are generally expected to refrain from alcohol and other drugs [60]. Furthermore, people undergoing parole supervision may be subjected to testing on suspicion that they have consumed these substances and detection of alcohol above 0.05 g per 100 milliliters (about one standard drink of alcohol) is considered a violation of their parole condition [60]. Thus, the need to refrain from these substances may make substance use a non-viable coping mechanism for this population. Furthermore, the need to appear compliant with parole conditions may have exacerbated social desirability and made participants not fully disclose their use of alcohol and other drugs. While social desirability could be a factor, and intuitively more so with regard to disclosure of sexual violence victimization, the risk related to disclosing substance use may have been judged by the participants to be higher in comparison. The use of substances by people undergoing parole supervision is a violation of their parole conditions which can result in them being given a written warning, being tested more frequently for substances, or being referred to court or the parole board [60].

## 5. Limitations

The study had some limitations that are worth noting. First, the sample size was small and thus compromised the power of the study to detect significant associations. Also, covariates (*e.g*., demographics such as age, education level, and relationship status, and incarceration-related factors such as previous conviction, duration of incarceration and gang affiliation) could not be included in the regression models because of the small sample size. These limitations highlight the need for further studies that are adequately powered. Second, the study’s cross-sectional design prevents the authors from drawing causal inferences from the findings. Third, some of the measures (*i.e*., prototypic and masculine depression, SV victimization and hypermasculinity measures) had not been used and/or validated for use with a South African sample prior to their use in this study. Validation of these instruments for the South African context should be taken up in future research. Finally, in the absence of existing norms or definitions of low, moderate and high scores for the hypermasculinity measure for our population, we relied on a convention that yields sample-specific values for these levels. The use of sample-specific values could present challenges for when comparing our findings with those of other similar studies.

## 6. Implications for practice and research

The findings in this study suggest that despite being sexually victimized, men who are high in hypermasculinity are less likely to experience prototypic depression than men who have average or low levels of hypermasculinity. Moreover, neither masculine depression nor alcohol and other drug use were associated with sexual victimization among the men in this study. While it may be that these findings demonstrate resilience among the men, it is more probable that hypermasculinity expectations of stoicism and a sense of invulnerability prevent the men who are high in hypermasculinity from identifying and/or expressing SV victimization-related emotional or psychological reactions. As such, a concern raised by the findings of this study is that if left unaddressed, over time the effects of the trauma (the SV victimization) can be expressed in destructive ways, including explosive anger, substance use, suicide and/or interpersonal violence [61]. Consequently, these findings suggest a need for gender transformative interventions and mental health promotion among men who have been sexually victimized. Gender transformative interventions involve engaging men in a process of critical reflection on gender roles and socialization. These interventions have been found useful in supporting men to construct health-promoting masculinities, including reductions in their risky behaviors (*e.g*., substance use) and improvements in their health seeking behaviors for mental health services [62, 63]. Mental health promotion facilitates mental health literacy and enables the achievement of positive mental health [64]. The combination of gender transformative interventions and mental health promotion could aim to sensitize incarcerated men and/or men with a history of incarceration to their emotional and internal states (*i.e*., promote gendered mental health literacy) and how the experience and expression of these, and related help-seeking behaviors may be influenced by prescriptive masculine norms [65]. Considering that SV victimization is itself under-reported, while masculine norms have been linked to other problems such as gang presence and interpersonal violence in correctional facilities [9, 66], such interventions could be delivered more widely within correctional settings such that they benefit men who have been sexually victimized and, at the same time, other incarcerated men. The identification of, and mental health care and support, for survivors of SV, must also be extended to community situated facilities that provide parole supervision to previously incarcerated men who are conditionally released from incarceration. At these facilities survivors may be more forthcoming about their experiences of victimization as, unlike when confined to the same space with a perpetrator, they will not be in immediate danger of revictimization. Finally, given that gender insensitive health systems negatively impacts men’s access to treatment [22, 62], care should be taken to ensure that the mental health care services and mental health care providers, both at correctional facilities (during incarceration) and community correctional centers (post incarceration) are attuned to the needs, interests and preferences of men who are the target recipients of their services.

The findings of this study further highlight the need for continuous theoretical and empirical re-evaluation of how depression presents among men in correctional settings and how it can be better assessed and diagnosed. The language used to describe prototypic depressive symptoms may be a good starting point given that men often do not describe their experience as depression or feeling down, with some studies showing that men rather opt for language like “stress” or “life hassles” [22, 67]. Moreover, further theoretical and methodological engagement is needed with the concepts of masculine and masked depression given that, although theoretically useful, they are empirically problematic as these variants of depression are difficult to assess by virtue of being hidden or different from what is (proto) typically known as depression [22, 28].

## 7. Conclusions

This study found that previously incarcerated men who strongly identify with hypermasculinity are less likely to show typical signs of depression after experiencing sexual victimization. Further research is needed to determine whether this decreased likelihood of depression results from avoidance of vulnerability or learned resilience in response to masculine expectations. To effectively support previously incarcerated male survivors of sexual violence, interventions must consider their adherence to societal expectations of masculinity.

## Figures and Tables

**TABLE 1.** Demographic and incarceration-related factors of participants (n = 67).

Variable	Categories	Mean (SD)	N(%)†
Age		31.7 (8.80)	
Education	<grade 12		38 (56.7)
	≥grade 12		27 (40.3)
Relationship status	Single/never married		43 (64.2)
	Currently/previously partnered		19 (28.4)
Number of years incarcerated		3.8 (4.00)	
Criminal offense (type)	Violent crime*		27 (40.3)
	Property and other** crime		36 (53.7)
Previous conviction	Yes		27 (40.3)
Involved in physical fight			30 (44.8)
Gang affiliation			19 (28.4)
Problem alcohol consumption (AUDIT)			32 (47.8)
Drug use (DUDIT)			16 (23.9)
Sexual Violence Victimization	At least once		14 (20.9)

† Categories may not add up to 100% due to missing values.

* Violent crime includes armed theft/housebreaking/burglary (n = 1) and attempted/completed assault, rape and murder (n = 21), possession of child pornography (n = 1), culpable homicide (n = 2) animal cruelty (n = 2).

** Property crimes include unarmed theft/housebreaking/burglary and fraud (n = 33); other crime includes possession of drugs (n = 1), contempt of court (n = 1), defeating the ends of justice (n = 1). CI: Confidence interval; SD: Standard deviation; AUDIT: Alcohol Use Disorder Identification Test; DUDIT: Drug Use Disorder Identification Test.

**TABLE 2.** Pearson’s correlation matrix of focal predictor (sexual violence victimization), moderator (hypermasculinity) and dependent variables (prototypic depression, masculine depression, alcohol use, drug use).

	(Low) Hypermasculinity		Sexual violence victimization	
	*r*	*p*	*r*	*p*
Sexual violence victimization	0.165	0.195		
Prototypic depression	0.387	0.002	−0.103	0.422
Masculine depression	0.286	0.023	0.017	0.894
Alcohol use	−0.209	0.099	0.160	0.210
Drug use	−0.165	0.196	−0.040	0.758

**TABLE 3.** Results of regression analysis of the moderation effect of hypermasculinity on the relationship between SV victimization and mental health outcomes.

Outcome	Variables		Coeff. (95% CIs)	*t*	*p*
Prototypic	Depression				
	Constant	*i*_*y*_	69.318 (64.130–74.506)	27.710	<0.001
	SV victimization	*b*_1_	−9.916 (−21.962–2.129)	−1.646	0.105
	Hypermasculinity	*b*_2_	0.296 (0.142–0.449)	3.852	<0.001
	SV victimization × (Low) Hypermasculinity	*b*_3_	0.339 (0.019–0.660)	2.118	0.038
*R*^2^ = 0.249, *F*(3, 62) = 4.949, *p* = 0.004					
Δ*R*^2^ = 0.041, *F*(1, 62) = 4.486*, p* = 0.038					
Masculine	Depression				
	Constant	*i*_*y*_	22.802 (21.310–24.293)	30.572	<0.001
	SV victimization	*b*_1_	0.096 (−3.106–3.298)	0.060	0.952
	Hypermasculinity	*b*_2_	0.057 (0.011–0.103)	2.475	0.016
	SV victimization × (Low) Hypermasculinity	*b*_3_	0.107 (0.019–0.194)	2.442	0.019
*R*^2^ = 0.140, *F*(3, 61) = 2.701, *p* = 0.053					
Δ*R*^2^ = 0.054, *F*(1, 61) = 5.963, *p* = 0.019					
Alcohol use					
	Constant	*i*_*y*_	7.715 (6.049–9.382)	9.261	<0.001
	SV victimization	*b*_1_	3.383 (−0.823–7.588)	1.609	0.113
	Hypermasculinity	*b*_2_	−0.038 (−0.076–0.000)	−1.998	0.050
	SV victimization × (Low) Hypermasculinity	*b*_3_	0.026 (−0.063–0.114)	0.578	0.565
*R*^2^ = 0.067, *F*(3, 60) = 2.889, *p* = 0.043					
Δ*R*^2^ = 0.003, *F*(1, 60) = 0.335, *p* = 0.565					
Drug use					
	Constant	*i*_*y*_	5.892 (−2.779–8.985)	3.789	<0.001
	SV victimization	*b*_1_	−1.040 (−11.396–9.316)	−0.201	0.842
	Hypermasculinity	*b*_2_	−0.051 (−0.144–0.041)	−1.107	0.273
	SV victimization × (Low) Hypermasculinity	*b*_3_	−0.052 (−0.341–0.237)	−0.361	0.720
*R*^2^ = 0.030, *F*(3, 62) = 0.660, *p* = 0.580					
Δ*R*^2^ = 0.004, *F*(1, 62) = 0.130, *p* = 0.720					

Coeff: Coefficient; CIs: Confidence Intervals; SV: sexual violence; ×: the interaction between the variables.

## Data Availability

The data presented in this study are available upon reasonable request from the corresponding author.

## References

[1] Berg MK, Hobkirk AL, Joska JA, Meade CS. The role of substance use coping in the relation between childhood sexual abuse and depression among methamphetamine users in South Africa. Psychological Trauma. 2017; 9: 493–499. 27710005 10.1037/tra0000207 PMC5501765

[2] Dworkin ER, Menon SV, Bystrynski J, Allen NE. Sexual assault victimization and psychopathology: a review and meta-analysis. Clinical Psychology Review. 2017; 56: 65–81. 28689071 10.1016/j.cpr.2017.06.002 PMC5576571

[3] Sampsel H Long-term mental and physical health outcomes for male victims of unwanted sexual violence: a systematic review. 2016. Available at: https://kb.osu.edu/items/f45a8ca3-4c3b-586a-8d18-819994f8c420 (Accessed: 23 October 2023).

[4] Hensley C, Koscheski M, Tewksbury R. Examining the characteristics of male sexual assault targets in a southern maximum-security prison. Journal of Interpersonal Violence. 2005; 20: 667–679. 15851535 10.1177/0886260505276069

[5] Papadakaki M, Tzamalouka GS, Chatzifotiou S, Chliaoutakis J. Seeking for risk factors of intimate partner violence (IPV) in a Greek national sample: the role of self-esteem. Journal of Interpersonal Violence. 2009; 24: 732–750. 18463309 10.1177/0886260508317181

[6] Struckman-Johnson C, Struckman-Johnson D. Sexual coercion rates in seven Midwestern prison facilities for men. The Prison Journal. 2000; 80: 379–390.

[7] Adam S Male rape in South African prisons. 1st edn. GRIN Verlag: Munich. 2011.

[8] Elena G, Marianne B, Dean P, Jean R, Raoul S. Stop prison rape in South Africa. Agenda. 2007; 21: 68–80.

[9] Gear S Behind the bars of masculinity: male rape and homophobia in and about south African men’s prisons. Sexualities. 2007; 10: 209–227.

[10] Javaid A Male rape, masculinities, and sexualities. International Journal of Law, Crime and Justice. 2018; 52: 199–210.

[11] Widanaralalage BK, Hine BA, Murphy AD, Murji K “I didn’t feel I was a victim”: a phenomenological analysis of the experiences of male-on-male survivors of rape and sexual abuse. Victims & Offenders. 2022; 17: 1147–1172.

[12] PettyJohn ME, Reid TA, Cary KM, Greer KM, Nason JA, Agundez JC, “I don’t know what the hell you’d call it”: a qualitative thematic synthesis of men’s experiences with sexual violence in adulthood as contextualized by hegemonic masculinity. Psychology of Men & Masculinities. 2022; 24: 272–290.

[13] Gear S, Ngubeni K. Daai ding: sex, sexual violence and coercion in men’s prisons. 1st edn. Centre for the study of violence and reconciliation: Johannesburg, South Africa. 2002.

[14] Ron Hugo. Talking about male rape: two narratives. Agenda. 2013; 27: 84–89.

[15] Foster S, Gul P, Bock JE. Masculine honor endorsement is linked with stigmatization of men who have been sexually assaulted. To be published in Psychology of Men & Masculinities. 2023. [Preprint].

[16] Thomas JC, Kopel J. Male victims of sexual assault: a review of the literature. Behavioral Sciences. 2023; 13: 304. 37102818 10.3390/bs13040304 PMC10135558

[17] Gear S Sexual violence in South African men’s prisons: causes, consequences and promising practices. In Aggleton (ed.) Routledge handbook of sexuality, gender, health and rights (pp. 10). 2nd edn. Routledge: Oxon, New York. 2023.

[18] Gear S. Fear, violence and sexual violence in a Gauteng juvenile correctional centre for males. (Briefing Report No 2). Centre for the study of violence and reconciliation: Johannesburg, South Africa. 2007.

[19] World Health Organisation. Suicide worldwide in 2019: global health estimates. World Health Organization: Geneva. 2021.

[20] Naidoo S, Mkize DL. Prevalence of mental disorders in a prison population in Durban, South Africa. African Journal of Psychiatry. 2012; 15: 30–35. 22344760 10.4314/ajpsy.v15i1.4

[21] Naidoo S, Subramaney U, Paruk S, Ferreira L. Mental illness and HIV amongst female inmates in Durban, South Africa. The South African Journal of Psychiatry. 2022; 28: 1628. 35169507 10.4102/sajpsychiatry.v28i0.1628 PMC8832006

[22] Addis ME, Hoffman E. Men’s depression and help-seeking through the lenses of gender. In Levant RF, Wong YJ (eds.) The psychology of men and masculinities (pp. 171–196). American Psychological Association: Washington, DC. 2017.

[23] Cleary A, Griffith DM, Oliffe JL, Rice S. Editorial: men, mental health, and suicide. Frontiers in Sociology. 2023; 7: 1123319. 36726599 10.3389/fsoc.2022.1123319 PMC9885186

[24] Kootbodien T, Naicker N, Wilson KS, Ramesar R, London L. Trends in suicide mortality in South Africa. 1997 to 2016. International Journal of Environmental Research and Public Health. 2020; 17: 1850. 32178393 10.3390/ijerph17061850 PMC7142470

[25] Bantjes J, Kagee A, Meissner B. Young men in post-apartheid South Africa talk about masculinity and suicide prevention. South African Journal of Psychology. 2017; 47: 233–245.

[26] von Zimmermann C, Hübner M, Mühle C, Müller CP, Weinland C, Kornhuber J, Masculine depression and its problem behaviors: use alcohol and drugs, work hard, and avoid psychiatry! European Archives of Psychiatry and Clinical Neuroscience. 2024; 274: 321–333. 36855002 10.1007/s00406-023-01567-0 PMC10914846

[27] Rice SM, Kealy D, Seidler ZE, Oliffe JL, Levant RF, Ogrodniczuk JS. Male-type and prototypal depression trajectories for men experiencing mental health problems. International Journal of Environmental Research and Public Health. 2020; 17: 7322. 33036428 10.3390/ijerph17197322 PMC7578926

[28] McDermott RC, Schwartz JP, Rislin JL. Men’s mental health: a biopsychosocial critique. In Wong YJ, Wester SR (eds.) APA handbook of men and masculinities (pp. 731–751). American Psychological Association: Washington, DC. 2016.

[29] Oliffe JL, Phillips MJ. Men, depression and masculinities: a review and recommendations. Journal of Men’s Health. 2008; 5: 194–202.

[30] UNODC. World Drug Report 2019. (United Nations publication, Sales No. E.19.XI.8). Vienna; June 19. 2019.

[31] World Health Organisation. Global status report on alcohol and health 2018. 2018. Available at: https://iris.who.int/bitstream/handle/10665/274603/9789241565639-eng.pdf?sequence=1 (Accessed: 23 October 2023).

[32] Pengpid S, Peltzer K, Ramlagan S. Prevalence and correlates of hazardous, harmful or dependent alcohol use and drug use amongst persons 15 years and older in South Africa: results of a national survey in 2017. African Journal of Primary Health Care & Family Medicine. 2021; 13: e1–e8. 10.4102/phcfm.v13i1.2847 PMC8008028 33764134

[33] South African Community Epidemiology Network on Drug Use. SACENDU full report: monitoring alcohol, tobacco and other drug abuse treatment admissions in South Africa, phase 51 (July–December 2021). 2023. Available at: https://www.samrc.ac.za/sites/default/files/attachments/2023-07/SACENDUFullReportPhase51.pdf (Accessed: 23 October 2023).

[34] Mutai KK, Stone J, Scheibe A, Fraser H, Johnson LF, Vickerman P. Trends and factors associated with illicit drug use in south Africa: findings from multiple national population-based household surveys, 2002–2017. International Journal of Drug Policy. 2024; 125: 104352. 38367327 10.1016/j.drugpo.2024.104352

[35] Cavanagh A, Wilson CJ, Kavanagh DJ, Caputi P. Differences in the expression of symptoms in men versus women with depression: a systematic review and meta-analysis. Harvard Review of Psychiatry. 2017; 25: 29–38. 28059934 10.1097/HRP.0000000000000128

[36] Connell RW. Masculinities. 2nd edn. Polity Press: Berkely. 2005.

[37] Jewkes R, Morrell R, Hearn J, Lundqvist E, Blackbeard D, Lindegger G, Hegemonic masculinity: combining theory and practice in gender interventions. Culture, Health & Sexuality. 2015; 17: 112–127. 10.1080/13691058.2015.1085094 PMC4706037 26680535

[38] Morse SJ, Wright KA. Imprisoned men: masculinity variability and implications for correctional programming. Corrections. 2019; 7: 23–45.

[39] Michalski JH. Status hierarchies and hegemonic masculinity: a general theory of prison violence. The British Journal of Criminology. 2015; 57: 40–60.

[40] Republic of South Africa. Department of correctional services annual report 2018/2019. 2019. Available at: https://www.gov.za/sites/default/files/gcis_document/201911/dcs-annualreport-201819.pdf (Accessed: 23 October 2023).

[41] Correctional services in Gauteng province. Available at: https://static.pmg.org.za/docs/1999/minutes/991117correctGauteng.htm (Accessed: 18 February 2023).

[42] Republic of South Africa. Department of Correctional Services Annual report 2020/2021. 2021. Available at: http://www.dcs.gov.za/wp-content/uploads/2021/11/DCS-AR-202021-FINAL-SIGNED.pdf (Accessed: 23 October 2023).

[43] Sousa VD, Rojjanasrirat W. Translation, adaptation and validation of instruments or scales for use in cross-cultural health care research: a clear and user-friendly guideline. Journal of Evaluation in Clinical Practice. 2011; 17: 268–274. 20874835 10.1111/j.1365-2753.2010.01434.x

[44] Burk LR, Burkhart BR, Sikorski JF. Construction and preliminary validation of the auburn differential masculinity inventory. Psychology of Men & Masculinity. 2004; 5: 4–17.

[45] Wolff N, Shi J, Bachman R. Measuring victimization inside prisons: questioning the questions. Journal of Interpersonal Violence. 2008; 23: 1343–1362. 18309042 10.1177/0886260508314301

[46] Wolff N, Blitz CL, Shi J, Bachman R, Siegel JA. Sexual violence inside prisons: rates of victimization. Journal of Urban Health. 2006; 83: 835–848. 16937087 10.1007/s11524-006-9065-2 PMC2438589

[47] World Health Organization; Babor TF, Higgins-Biddle JC, Saunders JB, Monteiro MG. AUDIT: the alcohol use disorders identification test: guidelines for use in primary health care. 2nd edn. World Health Organization: Geneva. 2001.

[48] Morojele NK, Kekwaletswe CT, Nkosi S, Kitleli NB, Manda SO. Reliability and factor structure of the AUDIT among male and female bar patrons in a rural area of South Africa. African Journal of Drug and Alcohol Studies. 2015; 14: 23–35.

[49] Berman AH, Bergman H, Palmstierna T, Schlyter F. Evaluation of the drug use disorders identification test (DUDIT) in criminal justice and detoxification settings and in a Swedish population sample. European Addiction Research. 2005; 11: 22–31. 15608468 10.1159/000081413

[50] Hildebrand M The psychometric properties of the drug use disorders identification test (DUDIT): a review of recent research. Journal of Substance Abuse Treatment. 2015; 53: 52–59. 25682718 10.1016/j.jsat.2015.01.008

[51] Bowen P, Zhang RP. Psychometric properties of the drug use disorders identification test (DUDIT) and prevalence of drug use among SA site-based construction workers. Psychology, Health & Medicine. 2022; 21: 1–12. 10.1080/13548506.2022.2103160 35861743

[52] Magovcevic M, Addis ME. The masculine depression scale: development and psychometric evaluation. Psychology of Men & Masculinity. 2008; 9: 117–132.

[53] Hayes AF. Mediation, moderation, and conditional process analysis. Introduction to mediation, moderation, and conditional process analysis: a regression-based approach. The Guilford Press: New York, NY. 2013.

[54] Hayes AF, Cai L. Using heteroskedasticity-consistent standard error estimators in OLS regression: an introduction and software implementation. Behavior Research Methods. 2007; 39: 709–722. 18183883 10.3758/bf03192961

[55] Simpson PL, Reekie J, Butler TG, Richters J, Yap L, Grant L, Factors associated with sexual coercion in a representative sample of men in Australian prisons. Archives of Sexual Behavior. 2016; 45: 1195–1205. 26597645 10.1007/s10508-015-0653-7

[56] Centre for the Study of Violence and Reconciliation. Gender-based violence (GBV) in South Africa: a brief review. Centre for the Study of Violence and Reconciliation: Braamfontein. 2016.

[57] Hu J, Feng B, Zhu Y, Wang W, Xie J, Zheng X. Gender differences in PTSD: susceptibility and resilience. In Aida (ed.) Gender differences in different contexts (pp. 23). IntechOpen: Rijeka. 2017.

[58] Forde C, Duvvury N. Sexual violence, masculinity, and the journey of recovery. Psychology of Men & Masculinity. 2017; 18: 301–310.

[59] Laws B The return of the suppressed: exploring how emotional suppression reappears as violence and pain among male and female prisoners. Punishment & Society. 2018; 21: 560–577.

[60] Republic of South Africa. Revised procedure manual: Supervision (Unit 1–8). 2017. http://www.dcs.gov.za/wp-content/uploads/2019/01/Procedure-Manual.pdf (Accessed: 20 November 2023).

[61] River J, Flood M. Masculinities, emotions and men’s suicide. Sociology of Health & Illness. 2021; 43: 910–927. 10.1111/1467-9566.13257 33751613

[62] Colvin CJ. Strategies for engaging men in HIV services. The Lancet HIV. 2019; 6: e191–e200. 30777726 10.1016/S2352-3018(19)30032-3

[63] Dworkin SL, Fleming PJ, Colvin CJ. The promises and limitations of gender-transformative health programming with men: critical reflections from the field. Culture, Health & Sexuality. 2015; 17: 128–143. 10.1080/13691058.2015.1035751 PMC4637253 25953008

[64] Jané-Llopis E, Barry M, Hosman C, Patel V. Mental health promotion works: a review. Promotion & Education. 2005; 2: 9–25, 61, 67. 10.1177/10253823050120020103x 15966248

[65] Seaton CL, Bottorff JL, Jones-Bricker M, Oliffe JL, DeLeenheer D, Medhurst K. Men’s mental health promotion interventions: a scoping review. American Journal of Men’s Health. 2017; 11: 1823–1837. 10.1177/1557988317728353 PMC5675255 28884637

[66] Kupers TA. Rape and the prison code. Prison Masculinities. 2001; 111: 112.

[67] Chuick CD, Greenfeld Jay M., Greenberg Stefanie Teri, Shepard Samuel J., Sam V. A qualitative investigation of depression in men. Psychology of Men & Masculinity. 2009; 10: 302–313.

